# CSC^high^E-cadherin^low^ immunohistochemistry panel predicts poor prognosis in oral squamous cell carcinoma

**DOI:** 10.1038/s41598-024-55594-5

**Published:** 2024-05-08

**Authors:** Rafael Carneiro Ortiz, Nádia Ghinelli Amôr, Luciana Mieli Saito, Mariana Rodrigues Santesso, Nathália Martins Lopes, Rodrigo Fonseca Buzo, Angélica Cristina Fonseca, Gleyson Kleber Amaral-Silva, Raquel Ajub Moyses, Camila Oliveira Rodini

**Affiliations:** 1 Department of Biological Sciences, Bauru School of Dentistry, University of São Paulo, Bauru, São Paulo Brazil; 2 Post-Graduation Program in Rehabilitation Sciences, Hospital for Rehabilitation of Craniofacial Anomalies, University of São Paulo (HRAC/USP), Av. Octavio Pinheiro Brisolla, 9-75, Jardim Brasil, São Paulo, Brazil; 3 Department of Surgery and Orthopedics, Faculty of Medicine, São Paulo State University (UNESP), Botucatu, São Paulo 18618-687 Brazil; 4 Department of Oral Pathology, Piracicaba Dental School, University of Campinas, Piracicaba, Brazil; 5 Department of Head and Neck Surgery, LIM28, Clinical Hospital HCFMUSP, School of Medicine, University of São Paulo, São Paulo, Brazil

**Keywords:** Cancer stem cells, Epithelial-mesenchymal transition, Digital pathology, Immunohistochemistry, Oral cancer, Metastasis, Oral cancer, Predictive markers, Cancer stem cells, Tumour biomarkers, Oncogenesis

## Abstract

Identifying marker combinations for robust prognostic validation in primary tumour compartments remains challenging. We aimed to assess the prognostic significance of CSC markers (ALDH1, CD44, p75NTR, BMI-1) and E-cadherin biomarkers in OSCC. We analysed 94 primary OSCC and 67 metastatic lymph node samples, including central and invasive tumour fronts (ITF), along with clinicopathological data. We observed an increase in ALDH1^+^/CD44^+^/BMI-1^-^ tumour cells in metastatic lesions compared to primary tumours. Multivariate analysis highlighted that elevated p75NTR levels (at ITF) and reduced E-cadherin expression (at the tumour centre) independently predicted metastasis, whilst ALDH1^high^ exhibited independent predictive lower survival at the ITF, surpassing the efficacy of traditional tumour staging. Then, specifically at the ITF, profiles characterized by CSC^high^E-cadherin^low^ (ALDH1^high^p75NTR^high^E-cadherin^low^) and CSC^intermediate^E-cadherin^low^ (ALDH1 or p75NTR^high^E-cadherin^low^) were significantly associated with worsened overall survival and increased likelihood of metastasis in OSCC patients. In summary, our study revealed diverse tumour cell profiles in OSCC tissues, with varying CSC and E-cadherin marker patterns across primary tumours and metastatic sites. Given the pivotal role of reduced survival rates as an indicator of unfavourable prognosis, the immunohistochemistry profile identified as CSC^high^E-cadherin^low^ at the ITF of primary tumours, emerges as a preferred prognostic marker closely linked to adverse outcomes in OSCC.

## Introduction

Oral squamous cell carcinoma (OSCC) accounts for over 300,000 new cases each year and currently represents 5% of all cancers worldwide^1,2^. Despite promising advances in therapeutic approaches, the 5-year survival rate remains around 50%, mainly due to the presence of cervical lymph node metastasis^3–6^. The pathogenesis of primary tumours, along with the presence of metastasis, has been linked to the presence and maintenance of cancer stem cells (CSC), which constitute a small subpopulation of tumour cells characterized by slow but unlimited division. These CSC play a significant role in invasion, drug resistance, and surveillance^7–10^.

Numerous molecules have initially been utilized in the endeavour to identify and eradicate CSCs implicated in OSCC carcinogenesis, both in comparison to normal mucosae and following radio/chemotherapy^11^. Nevertheless, the precise combination of markers within primary tumour compartments, specifically before treatment, which can be clinically validated for prognostic purposes, remains to be comprehensively delineated. One of the primary markers utilized for CSC selection is CD44, a membrane protein that promotes cell adhesion, enhances migratory abilities and is associated with the stemness phenotype^12^. In addition to CD44, the ALDH1 enzyme has also been extensively linked to the CSC phenotype in tumour cells, given its versatility in roles such as detoxification, protection against oxidative stress, retinoid metabolism, and tissue development^13^. Recently, our research group semi-quantitatively scored the immunoexpression of CSC markers, ALDH1 and CD44, in OSCC samples within the invasive tumour front (ITF) and metastatic lymph nodes as a whole. We found that CD44 immunoexpression was a predictor of lymph node metastasis, whilst ALDH1^high^ immunostaining was associated with angiolymphatic invasion, suggesting that immunoexpression of both markers links the CSC phenotype with invasion and metastasis and, could play important roles in OSCC carcinogenesis^14^. We then realized that the identification of a set of CSC markers associated with a different biological process marker in a large number of surgical resection specimen tissues, using a semi-automatized analysis, would be crucial to determining the understanding of OSCC carcinogenesis and its prognostic value.

Due to the complex behaviour of CSC, several other markers intimately involved in carcinogenesis have been isolated and identified, especially p75NTR and BMI-1, two important molecules associated with stemness phenotype^15^. This complexity arises from p75NTR's role as an essential receptor that modulates neurotrophin signalling, impacting neuronal survival, development, plasticity, and axonal growth. Its functions have direct implications for perineural invasion by tumour cells, contributing to poor prognoses in several carcinomas, including OSCC^16^. Furthermore, an in vitro study using the OSCC cell lineage OM-1 indicated that p75NTR signalling is the primary molecule responsible for forming tumour spheres in a cell suspension culture, suggesting a p75NTR-dependent property in cancer stemness^17^. On the other hand, BMI-1 appears to be a significant target for cancer biology, regenerative medicine, and ageing-related research due to its involvement in epigenetic regulation, stem cell maintenance, cancer development, ageing, DNA repair, and immune system modulation. Additionally, the overexpression of ALDH1 and BMI-1 can contribute to the poor prognosis of OSCC by promoting treatment resistance and malignant transformation, respectively^18,19^.

Understanding how CSC maintain their phenotype is essential for the development of targeted therapies that can specifically eliminate these cells. They are often responsible for tumour metastasis from primary tumours, which is associated with poor prognosis in various human carcinomas. The metastatic process in tumour cells is complex and can vary depending on factors such as the cancer type, interactions with the tumour microenvironment, and the therapies employed^20^. While the factors that regulate these mechanisms are not yet fully understood, there is evidence suggesting a link between CSC and the epithelial-mesenchymal transition (EMT). EMT is a biological process characterized by the loss of cell polarity and cell–cell adhesion, enabling tumour cells to migrate beyond the primary tumour^21^. Amidst the spectrum of EMT markers, the aberration or loss of E-cadherin, a transmembrane glycoprotein pivotal for mediating cell–cell adhesion in epithelial tissues, stands out as a significant contributor to pathological conditions, particularly in the context of cancer invasion and metastasis^22^. A recent study by Meng et al.^23^, utilizing immunohistochemistry (IHQ) on paraffin-embedded breast cancer samples, underscored the independent predictive value of reduced E-cadherin expression at the ITF concerning nodal metastasis. Furthermore, this reduced expression was associated with worse recurrence-free survival and disease-free survival. These findings emphasize the prognostic importance of E-cadherin, prompting the necessity for future research to establish its staining protocol for clinical applications.

Although the precise mechanisms by which CSCs regulate EMT in various malignancies remain unclear, this process typically involves the down-regulation of E-cadherin and the up-regulation of mesenchymal proteins^24–27^. It is noteworthy that tumour cells undergoing EMT can also undergo a reversal process known as mesenchymal-to-epithelial transition (MET) during metastasis. This MET enables the formation of a secondary tumour with epithelial characteristics resembling those of the original tumour^28,29^.

These intricate processes underscore the dynamic nature of cancer progression and metastasis, underscoring the need for further research to enhance our understanding of CSC-related mechanisms of specific markers intimately involved in carcinogenesis. Thus, we conducted quantitative assessments of the immunoexpression levels of CSC markers, including ALDH1, CD44, p75NTR, and BMI-1, along with E-cadherin, in both OSCC primary tumours and metastatic lymph nodes through the utilization of IHC. These assessments were then correlated with clinicopathological data. Our primary objective was to gain insight into the roles of these molecules within different tumour compartments during the progression of OSCC. Furthermore, we sought to establish connections between these markers and patient survival, with the ultimate aim of identifying a set of CSC markers and E-cadherin that are associated with the poor prognosis often observed in OSCC.

## Materials and methods

### Patients

Ninety-four smoker OSCC patients, aged 38 years or older, with no systemic diseases, and without any previous treatment before surgical resection and neck dissection surgery, were included. Paraffin-embedded samples consisted of 67 primary metastatic and 27 non-metastatic tumours, along with 67 cervical lymph node metastases, 47 of which originated from corresponding primary tumours. These samples correspond to archived material, spanning from January 2003 to December 2011.

### Immunohistochemistry technique and analysis

Sections were deparaffinized with xylene and then rehydrated using an ethanol series. Tissue antigens were retrieved as described previously^14^. Slides were incubated with primary anti-human ALDH1 (1:700, clone 44, cat# 611,195, BD Biosciences, NJ, USA), CD44 (1:700, clone 156-3C11, cat#3570, Cell Signalling, MA, USA) and E-cadherin antibodies (1:200, clone NCH-38, cat#M3612, DAKO, Carpinteria, CA) at room temperature (RT) for one hour. Overnight incubation at 4º C was carried out for the following primary antibodies: anti-human p75NTR (1:500, clone ME20.4, cat# 05–446, EMD Millipore, MA, USA) and BMI-1 antibodies (1:300, clone F6, cat#05–637, Merck, Darmstadt, Germany). Slides were further incubated with a biotin-free peroxidase-based ADVANCE™ kit (#K4068, DAKO) visualization system for one hour at RT, except for the BMI-1 antibody, which was performed using EnVision™ + Dual Link System-HRP (DAKO) for one hour at RT. The immunocomplexes were detected with 3´3-diaminobenzidine (DAB) substrate-chromogen system (DAKO) for one minute and counterstained with Meyer’s hematoxylin. The positive controls for immunostaining standardization were breast cancer cells (ALDH1), lymphocytes from cervical lymph nodes (CD44), testicle (p75NTR), and inflammatory fibrous hyperplasia (BMI-1 and E-cadherin). A negative control was obtained by incubation with IgG serum as a substitution for the primary antibodies^14^.

Slides were further scanned into high-resolution images using the Aperio Scanscope CS Slide Scanner (Aperio Technologies Inc, Vista, CA, USA). BMI-1 nuclear marker was analyzed using the Nuclear V9 algorithm (Aperio Technologies Inc), while cytoplasmic and membrane expression of ALDH1, CD44, p75NTR, and E-cadherin were analyzed by algorithm PixelCount V9 (Aperio Technologies Inc). For the markers analyzed by the algorithm PixelCount V9, each category received an intensity score: 1 to weak, 2 to moderate, and 3 to strong staining (Supplementary Fig. 1). The final score of each tumour was calculated as the sum of the percentage of each category multiplied by their intensity scores and, results always ranged from 100 to 300 based on the previous set of parameters protocoled^30^.

For quantification, immunopositivity tumour cells were counted in eighteen randomly distributed microscopic fields from each primary OSCC sample, nine from the centre regions of the tumour, and nine from the ITF (defined as the deepest field with infiltration of tumour cells). At metastatic sites, three microscopic fields randomly distributed in central and extracapsular areas presenting tumour cells were analysed. Then, eighteen of non-metastatic tumours (N0), and twenty-one of locoregional metastatic tumours (N +) standardized microscopic fields (50.000 µm^2^ rectangles each) were captured per slide.

### Validation of CSC markers and E-cadherin using public datasets

In this study, we conducted a comprehensive evaluation of CSC and E-cadherin gene expression patterns of human primary OSCC and metastatic lymph nodes by harnessing publicly accessible datasets. The acquisition of pertinent data was accomplished through a meticulous selection of validated resources. Firstly, we utilized the National Center for Biotechnology Information (NCBI) Database, with a specific focus on the Gene Expression Omnibus (GEO) repository (http://www.ncbi.nlm.nih.gov/geo/). Within the GEO repository, our attention was directed towards the GSE2280 messenger RNA (mRNA) microarray dataset. Furthermore, we expanded our data acquisition strategy to encompass the Xena Functional Genomics Explorer, affiliated with the University of California, Santa Cruz (https://xenabrowser.net/).

This study employed the limma (Linear Models for Microarray Analysis) package within the R statistical environment to identify genes differentially expressed (DE) between two or more experimental groups. This software leverages linear modelling techniques to assess gene expression variability while accounting for experimental design factors, providing robust statistical inference for DE analysis. Microarray data were pre-processed using appropriate normalization techniques to correct for systematic biases and ensure data comparability across samples. Linear models were constructed using limma, incorporating relevant experimental design factors as fixed effects. Moderated t-statistics were employed to estimate gene-wise expression differences between groups, accounting for potential variability within each group. To control for false positives arising from multiple comparisons, Benjamini–Hochberg adjusted p-values were calculated for each gene. Genes were considered differentially expressed if they met both a significance threshold (adjusted *p*-value < 0.05) and a fold change threshold (absolute fold change > 2). This stringent filtering strategy prioritizes genes exhibiting substantial expression changes likely to hold biological relevance within the context of our study.

Here, we directed our attention towards the Genomic Data Commons (GDC) of The Cancer Genomic Atlas (TCGA) Head and Neck Cancer (HNSC) dataset. As with the GEO dataset, our dataset selection criteria remained consistent, rigorously emphasizing the inclusion of validated studies featuring primary OSCC specimens and metastatic lymph nodes, along with accompanying clinicopathological information. This meticulous selection of publicly available datasets, each aligned with the established criteria, underpinned the robustness and validity of our data acquisition, forming the foundation for our comprehensive investigation of gene expression patterns in the context of OSCC and its metastatic potential for determining prognosis^55^.

### Statistics

Statistical analyses were performed using GraphPad Prism 8 (GraphPad Software, Inc., CA, USA) and JMP v11 (SAS, NC, USA). Protein immunoexpression was quantitatively compared between groups using the One-way ANOVA test and Dunn's multiple comparisons test. To establish dichotomous cut-off values distinguishing high and low immunostaining, we calculated the mean final score for each marker to characterize the evaluated sample regions. Stepwise logistic regression was used to find the best-fit model to predict the probability of lymph node metastasis and survival in OSCC, based on the immunostaining of CSC and E-Cadherin biomarkers both as numerical (absolute) and dichotomized (categoric) data at the centre and ITF of primary OSCC. TNM stage was also included in the analysis to assess the incremental predictive value of biomarkers. Finally, univariate analysis of clinicopathological characteristics and protein immunostaining profiles was conducted using Fisher's exact test. Missing data were imputed using the mean values of immunostaining in both the centres and ITF regions of primary tumours and whole metastatic lymph nodes. Two-tailed *p*-values < 0.05 were considered for statistical significance. For validating bioinformatics analysis, Benjamini & Hochberg (False discovery rate) was used to establish an adjusted *p*-value.

### Ethics approval and patient consent statement

This retrospective study was performed following the Declaration of Helsinki and approved by the Research Ethics Committee of Bauru School of Dentistry—University of Sao Paulo (Protocol CAAE 50,695,815.2.0000.54.17). The requirement to obtain written informed consent was waived by the Research Ehtics Committee for Project Reviews at the Clinical Hospital of the University of Sao Paulo Medical School from the Department of Head and Neck Surgery.

## Results

### Sample characterization

Demographic data, tumour location, and stage for the 94 patients enrolled are summarized in Table 1. Most OSCC patients were male (n = 81; 86.17%), aged from 38 to 86 years old, predominantly younger than 60 years old (n = 65; 69.15%) and all patients were smokers and drinkers. Microscopically, OSCC predominantly exhibited perineural invasion (n = 54; 57.45%) but no blood and lymphatic invasion (n = 70; 74.47%; n = 57; 60.64%, respectively). Also, the peritumoral inflammatory infiltrate was mostly moderate (n = 37; 39.36%) followed by a low density of inflammatory cells (n = 34; 36.17%). Following TNM (UICC)^31^ and WHO^32^ grading systems for OSCC, the majority of the lesions were T4 (n = 29; 30.85%) and T2 (n = 27; 28.72%), as well as N + (n = 64; 68.09%), exhibiting a moderate (n = 52; 55.32%) and well- (n = 36; 38.30%) patterns of differentiation. According to the 8^th^ Stage edition, most patients were in the IV stage (n = 59; 62.77%), and among all patients who died, most had a survival rate of fewer than 5 years (n = 44; 46.81%).

**Table 1:** Clinicopathological features of OSCC patients (n = 94 patients).

Characteristics	Category	N	(%)
Age	< 60	65	69,15
	≥ 60	29	30,85
Gender	Female	13	13,83
	Male	81	86,17
T category	T1	21	22,34
	T2	29	30,85
	T3	13	13,83
	T4	29	30,85
	Unavailable	2	2,13
N category	N0	26	27,66
	N +	64	68,09
	Unavailable	2	2,13
Perineural invasion	Yes	54	57,45
	No	35	37,23
	Unavailable	5	5,32
Blood vessel invasion	Yes	19	20,21
	No	70	74,47
	Unavailable	5	5,32
Lymphatic invasion	Yes	21	22,34
	No	57	60,64
	Unavailable	16	17,02
Staging 8^th^	I	2	2,13
	II	8	8,51
	III	23	24,47
	IV	59	62,77
	Unavailable	2	2,13
Peritumoural inflammatory infiltrate	Weak	34	36,17
	Moderate	37	39,36
	Intense	8	8,51
	Unavailable	15	15,96
Histological grade	Well	36	38,30
	Moderate	52	55,32
	Poorly	6	6,38
Survival	< 5 years	44	46,81
	≥ 5 years	23	24,47

Patients were staged according to the TNM Classification of Malignant Tumours staging manual.

### Immunohistochemical labelling for CSC markers—ALDH1, CD44, p75NTR and BMI-1 and E-cadherin in OSCC and cervical lymph node metastasis

**Figure 1:**
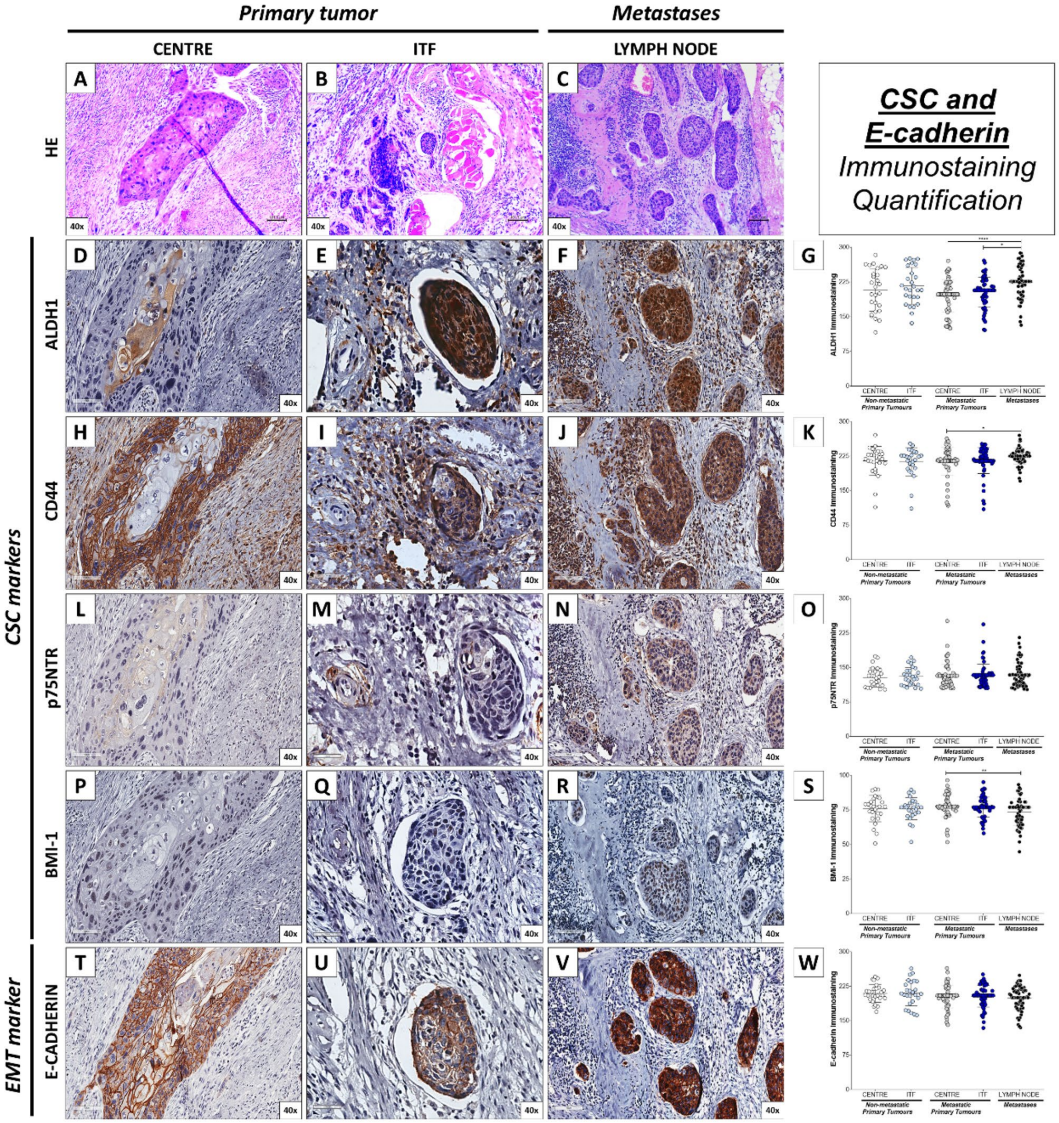
Immunohistochemistry of CSC markers and E-cadherin in OSCC (Centre and ITF) and cervical lymph node metastasis. (**A**) Central regions of primary tumours display expansive areas with an adherent cell-like structural architecture. (**B**) The ITF features more invasive cells, often observed individually or in small clusters, and occasionally associated with lymphatic emboli. (**C**) Lymph node metastases exhibit distinct histological features compared to central regions, especially within extracapsular areas. (**D**) ALDH1 immunostaining in central tumour areas typically manifests as sporadic cytoplasmic staining, predominantly encircling corneal pearls. (**E**) ALDH1-positive tumour cells exhibit increased immunostaining within lymphatic emboli. (**F**) Metastatic tumour cells display notably high levels of ALDH1 staining. (**G**) Statistical analysis reveals a significant increase in ALDH1-positive cells within metastatic sites compared to both central and ITF regions of primary tumours. (**H**) CD44 demonstrates homogeneous immunostaining in central regions. (**I**) Uniform immunostaining within the ITF of primary tumours for CD44. (**J**) CD44 displays increased immunostaining at metastatic sites. (**K**) Notably higher CD44 immunostaining levels are observed at metastatic sites compared to central regions of primary tumours. (**L**) p75NTR presence is predominantly detected in metastatic primary tumours. (**M**) Limited and scattered expression of p75NTR in central regions. (**N**) Strong cytoplasmic staining for p75NTR is evident in metastatic sites. (**O**) No statistical significance in p75NTR expression is observed among the groups. (**P**) Focal nuclear immunostaining for BMI-1 is predominantly observed in both central regions. (**Q**) BMI-1 focal nuclear immunostaining is also observed in the ITF of primary tumours. (**R**) Positive BMI-1 staining is detected in only a few scattered metastatic tumour cells. (**S**) The majority of BMI-1 immunopositivity significantly decreases in metastatic sites compared to central areas of primary tumours. (**T**) E-cadherin exhibits strong and consistent membrane intensity in the central regions. (**U**) Heterogeneous cytoplasmic positivity for E-cadherin is observed in invasive tumour cells within lymphatic emboli. (**V**) Lymph node metastasis shows strong positivity for E-cadherin. (**W**) No significant differences in E-cadherin expression are observed among the groups.

The central regions of primary tumours were primarily characterized by expansive areas populated by tumour cells exhibiting an adherent cell-like structural architecture, which varies according to tumour grade (Fig. 1A). Conversely, the ITF is distinguished by the presence of more invasive cells, often found either individually or in small clusters, some of which invade blood and lymphatic vessels (Fig. 1B), as well as perineural spaces. Remarkably, lymph node metastases exhibit distinct histological features, demonstrating disparities between central regions and extracapsular areas. In lymph nodes, it is possible to observe groups of tumour cells surrounded by lymphoid tissue (Fig. 1C). Notably, primary tumours generally displayed a consistent pattern of CSC panel staining, except for E-cadherin, which exhibited a contrasting profile. Specifically, in terms of the evaluated markers, ALDH1 immunostaining at the tumour centre areas typically manifested as sporadic cytoplasmic staining, predominantly encircling corneal pearls (Fig. 1D), whereas it was more pronounced in the expression observed in the more invasive cells found within lymphatic emboli (Fig. 1E) and metastatic tumour cells (Fig. 1F). Our observations further revealed that the final score (mean rank difference) of ALDH1-positive cells displayed a statistically significant increase in metastatic sites compared to primary tumours, both within the central and ITF regions (*p* < 0.0001 and *p* = 0.0156, respectively; Fig. 1G).

Conversely, CD44 exhibited widespread expression in the membrane and transmembrane regions of tumour cells across all sites (Fig. 1H,I). While CD44 immunostaining was increased at metastatic sites (Fig. 1J), it was notably significantly higher than that observed in central regions of primary tumours (*p* = 0.0463; Fig. 1K). For p75NTR, its presence was mostly found in metastatic primary tumours, showed limited and scattered expression (Fig. 1L) in centre and ITF (Fig. 1L,M, respectively), but strong cytoplasmatic staining in metastases (Fig. 1N), although without statistical significance (Fig. 1O). Focal nuclear immunostaining for BMI-1 was also observed mostly in both regions of primary tumours (Fig. 1P,Q), with positive tumours detected only in a few and scratched metastatic tumour cells (Fig. 1R). These results demonstrated that the majority of BMI-1 immunopositivity significantly decreased in metastatic sites compared to centre areas of primary tumours (*p* = 0.0052; Fig. 1S). Furthermore, E-cadherin showed strong consistent membrane intensity in the central regions (Fig. 1T), displayed heterogeneous cytoplasmatic positivity in invasive tumour cells within lymphatic emboli (Fig. 1U), and lymph node metastasis (Fig. 1V) displayed strong positivity for E-cadherin. However, it did not present significant differences among groups (*p* > 0.05; Fig. 1W).

### Immunostaining panel for OSCC poor prognosis

**Figure 2:**
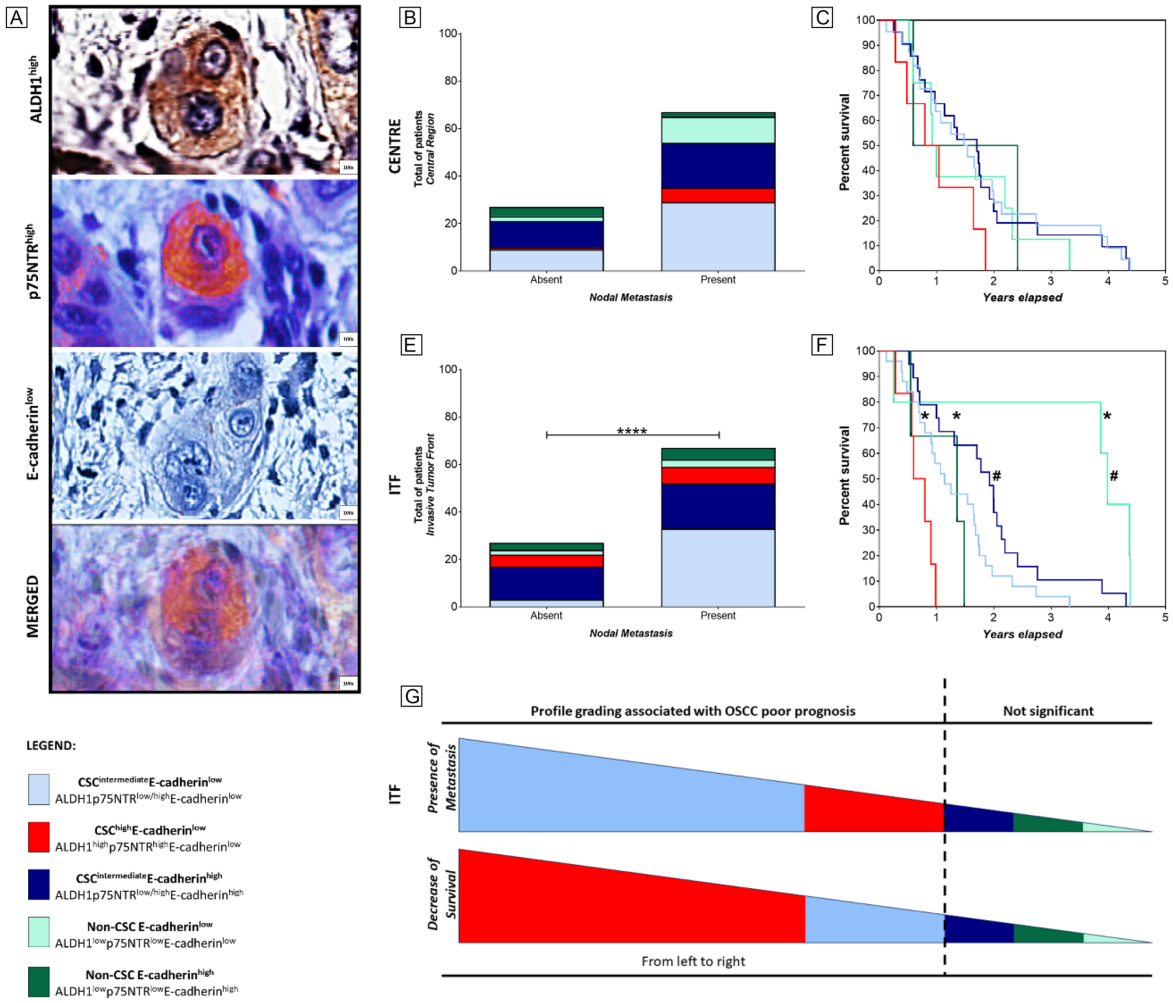
CSC model for OSCC poor prognosis. (**A**) Computerized staining co-localization for CSC^high^E-cadherin^low^ (ALDH1^high^p75NTR^high^E-cadherin^low^). (**B**, **C**) Tumour cell immune profiles at the centre of primary tumours had no impact on prognosis (*p* > 0.05). (**D**) CSC^intermediate^ E-cadherin^low^, followed by the CSC^high^ E-cadherin^low^ immune profile at the ITF of primary OSCC were associated with nodal metastasis (*p* = 0.0179). (**E**) CSC^high^ E-cadherin^low^ profile was the strongest predictor of poor 5-year overall survival, compared to CSC^intermediate^ E-cadherin^low^ (*p* = 0.0082), Non-CSC E-cadherin^low^ (*p* = 0.0002), and Non-CSC E-cadherin^high^ (*p* = 0.0254) profiles. The CSC^intermediate^ E-cadherin^low^ profile also demonstrated lower 5-year overall survival compared to CSC^intermediate^ E-cadherin^high^ (*p* = 0.0374), and Non-CSC E-cadherin^low^ (*p* = 0.0019). (**F**) shows the immune profile grading according to the presence of metastasis and survival decrease; from left to right is the most to the least related profile associated with OSCC's poor prognosis. * Means significant associations between CSC^high^ E-cadherin^low^ profile with others, whilst # means significant associations between CSC^intermediate^ E-cadherin^low^ profile with others.

Multivariate analysis was performed to ascertain poor prognostic factors associated with the presence of metastasis and diminished survival rates (specifically, 1-year and 5-year survival rates) in patients with OSCC. E-cadherin^low^ at the centre of primary OSCC and p75NTR^high^, both categoric and absolute, in ITF of primary OSCC were independent predictors of lymph node metastasis. ALDH1^high^ immunostaining at the ITF was a predictor of a decrease in 1-year overall survival in primary OSCC. Notable, ALDH1, p75NTR, and E-cadherin emerged as independent prognostic indicators in OSCC, exhibiting greater predictive efficacy compared to the conventional gold standard of tumour staging (Tables 2 and 3). Consequently, the potential profile associated with poor prognosis in OSCC was identified as CSC^high^E-cadherin^low^ (ALDH1^high^p75NTR^high^E-cadherin^low^) (Fig. 2A).

**Table 2:** Logistic regression for cervical lymph node metastasis and survival in OSCC’s central regions.

Region	Variable	Group	Lymph Node Metastasis			1-year Survival		
			Odds ratio	HR (95% CI)	P	Odds ratio	HR (95% CI)	P
Centre	ALDH1	Absolute	0.043	0.0007–2.5588	0.123	5.507	0.0507–597.47	0.471
		Low–High	0.493	0.0680–3.5750	0.482	1.821	0.1942–17.085	0.599
	CD44	Absolute	1.597	0.0218–116.48	0.831	0.025	0.0001–3.3908	0.120
		Low–High	1.405	0.2560–7.7064	0.696	0.745	0.1068–5.1976	0.766
	p75NTR	Absolute	0.946	0.0079–112.92	0.982	206.6	0.0669–638,070	0.146
		Low–High	0.467	0.0877–2.4808	0.373	3.360	0.3234–34.909	0.290
	BMI-1	Absolute	41.550	0.7407–2330.6	0.065	0.893	0.0056–142.16	0.965
		Low–High	1.355	0.2645–6.9418	0.716	6.965	0.8758–55.402	0.059
	E-cadherin	Absolute	4.258	0.0517–350.57	0.523	12.55	0.1252–1258.2	0.285
		Low–High	7.333	1.2979–41.431	0.0191*	0.576	0.0820–4.0453	0.574
	Stage	I–III	0.611	0.2152–1.7373	0.351	2.648	0.7044–9.9581	0.131
		IV						

ALDH1—aldehyde dehydrogenase 1; CD44—cell surface adhesion receptor; p75NTR—p75 neurotrophic receptor; BMI-1—B cell-specific Moloney murine leukaemia virus integration site 1; ITF—Invasive tumour front; HR—Hazard Ratio; Stage I to III—tumour may have grown larger and may have spread to nearby lymph nodes but still has not metastasized to distant parts of the body. The extent of lymph node involvement and tumour size will determine the substage; Stage IV—The most advanced stage of oral squamous cell carcinoma. *Means statistically significant p-value.

**Table 3:** Logistic regression for cervical lymph node metastasis and survival in OSCC’s ITF.

Region	Variable	Group	Lymph Node Metastasis			1-year Survival		
			Odds ratio	HR (95% CI)	*P*	Odds ratio	HR (95% CI)	*P*
Invasive tumour front	ALDH1	Absolute	0.158	0.0030–8.0185	0.347	0.039	0.0003–4.3761	0.171
		Low–High	1.040	0.1727–6.2589	0.966	0.101	0.0108–0.9355	0.039*
	CD44	Absolute	9.484	0.2782–323.23	0.213	25.159	0.3725–1698.8	0.111
		Low–High	2.586	0.4493–14.883	0.285	1.823	0.2522–13.180	0.549
	p75NTR	Absolute	396.740	1.3099–20,157	0.021*	0.002	0.0000–9.2820	0.139
		Low–High	0.122	0.0173–0.8602	0.027*	2.286	0.1949–26.814	0.515
	BMI-1	Absolute	3.063	0.0285–328.59	0.638	12.143	0.0244–6024.4	0.419
		Low–High	0.545	0.0977–3.0388	0.485	0.738	0.0818–6.6556	0.787
	E-cadherin	Absolute	0.678	0.0078–58.808	0.864	1.627	0.0086–306.28	0.855
		Low–High	2.715	0.4592–16.046	0.268	6.650	0.6627–66.723	0.092
	Stage	I–III	0.5105	0.1707–1.5269	0.222	0.310	0.0789–1. 221	0.078
		IV						

ALDH1—aldehyde dehydrogenase 1; CD44—cell surface adhesion receptor; p75NTR—p75 neurotrophic receptor; BMI-1—B cell-specific Moloney murine leukaemia virus integration site 1; ITF—Invasive tumour front; HR—Hazard Ratio; Stage I to III—tumour may have grown larger and may have spread to nearby lymph nodes but still has not metastasized to distant parts of the body. The extent of lymph node involvement and tumour size will determine the substage; Stage IV—The most advanced stage of oral squamous cell carcinoma. *Means statistically significant p-value.

To substantiate this hypothesis, we conducted a rigorous validation by cross-referencing our findings with publicly accessible datasets. Subsequently, we classified the outcomes of each biomarker into distinct immunostaining profiles, taking into account their expression in both the central and ITF regions. The number distribution of profiles differed between the central regions of primary tumours and the ITF. In the primary tumour centre, the profiles were CSC^intermediate^E-cadherin^low^ (38, 40.4%), CSC^intermediate^E-cadherin^high^ (30, 31.9%), Non-CSC Epithelial^high^ (13, 13.8%), CSC^high^E-cadherin^low^ (7, 7.4%), and Non-CSC Epithelial^low^ (6, 6.3%). Controversially at the ITF, the profiles were CSC^intermediate^E-cadherin^low^ (36, 38.2%), CSC^intermediate^E-cadherin^high^ (33, 35.1%), CSC^high^E-cadherin^low^ (12, 12.7%), Non-CSC Epithelial^low^ (8, 8.5%), and Non-CSC Epithelial^high^ (5, 5.3%) (Table 4; Supplementary Fig. 2).

**Table 4:** CSC and E-cadherin profiles for OSCC prognosis.

	Source	Tumour site	Analysis	ALDH1	p75NTR	E-cadherin	Immunostaining profile	N (%)
PRIMARY OSCC	REGION	CENTRE	Immunostaining Pixel Count V9	high	high	low	CSC^high^E-cadherin^low^	7 (7.4)
				low/high	low/high	low	CSC^intermediate^E-cadherin^low^	38 (40.4)
				low/high	low/high	high	CSC^intermediate^E-cadherin^high^	30 (31.9)
				low	low	high	Non-CSC Epithelial^high^	13 (13.8)
				low	low	low	Non-CSC Epithelial^low^	6 (6.3)
		ITF		high	high	low	CSC^high^E-cadherin^low^	12 (12.7)
				low/high	low/high	low	CSC^intermediate^E-cadherin^low^	36 (38.2)
				low/high	low/high	high	CSC^intermediate^E-cadherin^high^	33 (35.1)
				low	low	high	Non-CSC Epithelial^high^	5 (5.3)
				low	low	low	Non-CSC Epithelial^low^	8 (8.5)
	Public database	GEO (LogFC)	Affymetrix Human Genome U133A Array	high	high	low	N.A	
		Xena Browser (LogFC)	Illumina—RNAseq	high	high	low		

In our comparative analysis, encompassing datasets from GEO and the Xena Browser, we observed consistent trends. Specifically, ALDH1 and p75NTR exhibited upregulation, while E-cadherin displayed a downregulation in primary OSCC cases that exhibited metastatic behaviour. This trend was also noted in metastatic lesions when compared to their respective primary tumours. However, it is noteworthy that these observed differences did not attain statistical significance, as indicated by adjusted *p*-values exceeding 0.05.

Univariate analyses aimed at predicting nodal metastasis and reduced overall survival did not yield statistically significant associations, as evidenced in Fig. 2B,C, respectively. Conversely, the most salient immunostaining profile for prognosticating a poor outcome emanated from the ITF, displaying variability. Regarding the presence of metastasis, the most significant association was with the CSC^intermediate^ E-cadherin^low^ immunostaining, followed by CSC^high^ E-cadherin^low^ (*p* = 0.0179; Fig. 2D). Concerning survival, the CSC^high^E-cadherin^low^ immunostaining profile was the strongest predictor of poor 5-year overall survival, although the CSC^intermediate^ E-cadherin^low^ profile also demonstrated lower 5-year overall survival compared to CSC^intermediate^ E-cadherin^high^ (*p* = 0.0374), and Non-CSC E-cadherin^low^ (*p* = 0.0019) (Fig. 2E). Figure 2F provides a schematic representation of the immunostaining profiles at the ITF related to metastasis and survival.

Thus, grouping the immunostaining levels of ALDH1, p75NTR, and E-cadherin was consistent with the outcomes from the multivariate analysis, validating that the CSC^high^E-cadherin^low^ and CSC^intermediate^ E-cadherin^low^ profiles can be used as predictors of worse overall survival and metastasis in OSCC. Remarkably, considering the significant decrease in overall survival as the worst clinical outcome, we may state that the CSC^high^E-cadherin^low^ is the preferred immunoprofile associated with a poor prognosis in OSCC.

## Discussion

The outcomes of our investigation propose a potential immunohistochemistry panel of biomarkers associated with an unfavourable prognosis in OSCC. This proposal is founded on the immunostaining levels of CSC and E-cadherin markers observed across distinct primary tumour compartments, all evaluated before any chemo/radio treatment. Initially, our findings revealed that tumour cells with ALDH1^high^, p75NTR^high^, and/or E-cadherin^low^ profile at the ITF were independent predictors of metastasis and lower 1-year survival. Later, we hypothesize that a collective CSC^high^E-cadherin^low^ immunostaining profile would correspond to the major subpopulation involved in OSCC poor prognosis. To validate this, we analysed the combination of ALDH, p75NTR, and E-cadherin immunopositivity for predicting poor prognosis in OSCC. We found that the CSC^high^E-cadherin^low^ and CSC^intermediate^ E-cadherin^low^ profiles were most significantly associated with nodal metastasis and overall survival decrease.

In the current investigation, we initially observed a significant elevation of ALDH1^+^ tumour cells in metastatic sites when compared to both the centre and ITF regions of primary tumours. Importantly, our multivariate analysis unveiled that heightened ALDH1 immunostaining in tumour cells within the ITF served as an independent predictor of diminished survival, collectively underscoring the substantial potential for ALDH1 as an important CSC driver in OSCC carcinogenesis, and progression. These findings align with our prior report, which demonstrated an association between the high presence of ALDH1 in tumour cells with the angiolymphatic invasion in primary OSCC^14^. Moreover, analogous studies have also reported important roles of ALDH1 in carcinogenesis and aggressive tumour progression, particularly concerning lower tumour grade differentiation, the presence of lymph node metastasis, reduced overall survival, and decreased disease-free survival^33,34^. These attributes collectively contribute to an unfavourable prognosis^35–37^. The ALDH1 enzyme also assumes responsibility for oxidizing a diverse array of intracellular aldehydes, and within malignant cells, it may be utilized to facilitate several intrinsic surveillance mechanisms aimed at enhancing migratory capabilities, self-renewal and drug resistance properties^38^.

Furthermore, recent research has shed light on the roles of both ALDH1 and CD44 as indicators of breast cancer malignancy, highlighting their distinct functions in tumorigenesis and progression. Notably, these investigators demonstrated that CD44 promotes self-renewal, proliferation, and tumour growth in xenograft mouse models, while ALDH1 enhances invasion and metastasis in vitro and in vivo, respectively^34^. CD44, recognized as a multifunctional cell surface adhesion molecule, exhibits diverse roles in carcinogenesis^39,40^. In our study, initial findings revealed elevated CD44 staining in metastatic sites compared to primary tumours, although multivariate analysis did not reveal a significant association with poor prognosis. It is well-established that subpopulations of CSC coexist and display plasticity, transitioning between preferentially migratory and proliferative phenotypes^41^. The present study's findings suggest that, while CD44 may not directly correlate with metastatic dissemination, its heightened presence in metastatic cells could be indicative of an increased migratory and proliferative status within the CD44^high^ subpopulation in OSCC. Accordingly, our research group previously demonstrated in a xenograft model that the CD44^high^ESA^high^ (Epi-CSC) subpopulation of LUC4 OSCC cells generated tumours more effectively and larger than the CD44^high^ESA^low^ (EMT-CSC) phenotype. Similarly, Epi-CSC displayed higher colony formation capacity and proliferative activity in vitro than EMT-CSC^42^. Boxberg et al.^43^ also documented that the upregulation of CD44 at the ITF in primary OSCC tumours exhibited correlations with poor histopathological differentiation, heightened tumour budding activity, and single-cell invasion. Significantly, the overexpression of CD44 in lymph node metastases emerged as an independent prognostic determinant associated with diminished overall survival, disease-specific survival, and disease-free survival in patients afflicted with advanced OSCC. Collectively, these findings underscore the notion that CD44^high^ tumour cells, while possessing augmented proliferative and tumorigenic potential, necessitate the involvement of EMT molecules to facilitate the migration and invasion of distant anatomical sites.

Numerous studies have also established a close association between p75NTR and the progression of OSCC, underlining its role in enhancing proliferation, invasion, and tumorigenicity across various human cancer types^44,45^. Notably, the expression of p75NTR, particularly at the ITF of OSCC, has been linked to reduced 5-year disease-free survival rates and an increased likelihood of recurrence, thus contributing significantly to the overall poor prognosis of OSCC^46^. Our findings are consistent with the existing literature, as heightened immunostaining of p75NTR in tumour cells within the ITF of primary OSCC emerged as a critical and independent predictor of nodal metastasis, as determined by multivariate analysis. This correlation can be rationalized by the observed co-expression of p75NTR with Ki-67, indicative of increased cellular proliferation capacity^47,48^. Nevertheless, it is well-documented that tumour cells possessing stemness properties within the ITF exhibit a heightened propensity for local metastasis through a myriad of extrinsic and intrinsic mechanisms^49^. Consequently, our findings substantiate the notion that p75NTR expression constitutes a pivotal step in the facilitation of effective metastasis formation and may serve as a valuable predictor of metastatic events. Still, in the context of CSC markers, we initially found a significant decrease in BMI-1 immunopositivity in nodal metastasis compared to primary tumours. Although the association of BMI-1 levels with OSCC poor outcome was not confirmed by multivariate analysis, it is known that BMI-1 is involved in cell cycle regulation, immortalization, and senescence^50^. We may suggest that our findings agree with the literature due to the frequent association of BM1-1^high^ expression with early carcinogenesis in OSCC. Also, other studies have supported the correlation between the lack of BMI-1 immunoexpression and with poor prognosis of OSCC patient^51,52^. It is known that the CSC phenotype is a transient state, that can be regulated by the tumour microenvironment and therapeutic pressures, leading to the hypothesis that BMI-1 may act in primary tumours providing plasticity in oral tumour cells, and inducing cell migration, but not enough to maintain this phenotype at metastatic colonies.

In contrast to the heightened expression of ALDH1 and p75NTR CSC markers, our investigation revealed that low immunostaining of E-cadherin emerged as a significant and independent predictor of metastasis within both the centre and ITF regions of primary tumours. These findings align with the established role of E-cadherin in governing tumour cell migration, as evidenced by the diminished or absent expression of E-cadherin in both individual and collective invasive tumour cells at the ITF. This downregulation contributes to the induction of EMT, ultimately facilitating the metastatic cascade^53^. Furthermore, prior research has underscored that E-cadherin downregulation, facilitated through HSPD1 activation, promotes the migration and invasion of OSCC tumour cells into adjacent organs, thus fostering metastatic dissemination and correlating with an unfavourable prognosis^54^. While the precise intricacies of the molecular interplay between CSCs and EMT remain incompletely elucidated, the association between these processes is pivotal for comprehending the behaviour of tumours.

**Figure 3:**
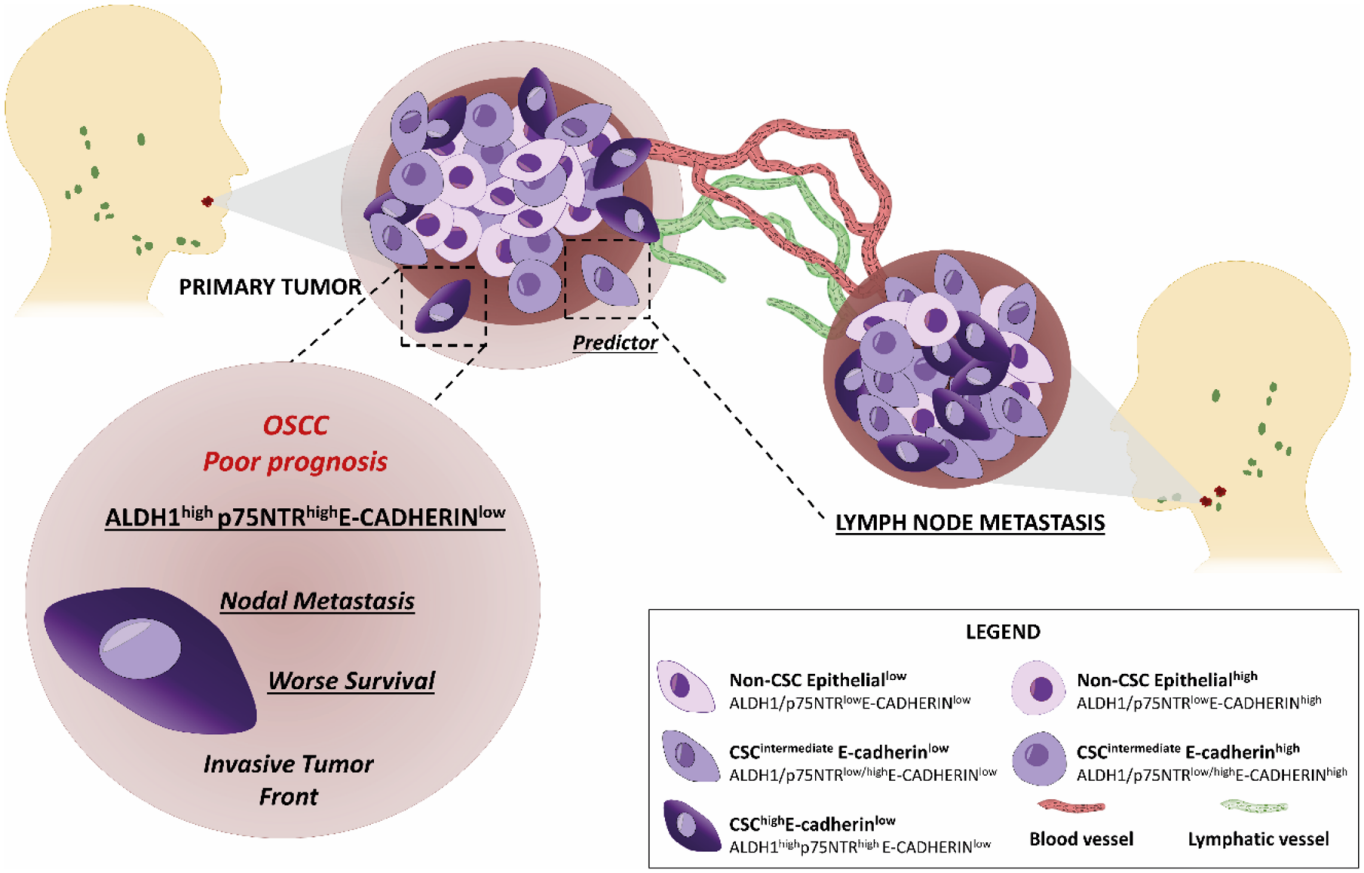
CSC markers and E-cadherin as a putative panel of poor prognosis in Oral Squamous Cell Carcinoma. (**A**) Tumour compartments, designed as centre and ITF, exhibited tumour cells with different patterns of CSC and E-cadherin immunopositivity (bottom right square). At the ITF, ALDH1^high^ and p75NTR^high^ immunostaining were independent predictors of decreased survival and the presence of metastasis, respectively. The most clinically relevant profile was the CSC^high^E-cadherin^low^ (ALDH1^high^p75NTR^high^E-cadherin^low^) at the ITF, which was a predictor of metastasis and lower overall survival and, could be used as a panel of biomarkers for poor prognosis in OSCC.

In conclusion, our study has revealed the existence of distinct tumour cell profiles within tissue samples of OSCC, characterized by variations in immunostaining levels of CSC and E-cadherin markers across primary tumours and metastatic sites. This association was in agreement with independent OSCC publically available datasets. Given that reduced survival rates serve as a pivotal indicator of a poor prognosis, the immunohistochemistry profile identified as CSC^high^E-cadherin^low^ at the ITF of primary tumours, before treatment, emerges as the preferred prognostic marker associated with unfavourable outcomes in OSCC. It is noteworthy that while CSC and E-cadherin markers have been extensively investigated independently within the context of OSCC, this study, to the best of our knowledge, represents the first substantial association of a distinct combined immunohistochemistry profile encompassing both CSC and E-cadherin markers with adverse prognostic implications in OSCC (Fig. 3).

### Supplementary Information

Supplementary Figure 1.

Supplementary Figure 2.

## Data Availability

The data that support the findings of this study are available from the corresponding author upon reasonable request.

## Abbreviations

CSC: Cancer stem cells
EMT: Epithelial-mesenchymal transition
OSCC: Oral squamous cell carcinoma
ALDH1: Aldehyde dehydrogenase 1
CD44: Cell surface adhesion receptor
p75NTR: P75 neurotrophic receptor
BMI-1: B cell-specific Moloney murine leukaemia virus integration site 1
ITF: Invasive tumour front
MET: Mesenchymal-to-epithelial transition
IHC: Immunohistochemistry technique
