# Supplementary material for: CSChighE-cadherinlow immunohistochemistry panel predicts poor prognosis in oral squamous cell carcinoma
Source: Sci Rep. 2024 May 8;14:10583. doi: 10.1038/s41598-024-55594-5 (PMC11078993; doi:10.1038/s41598-024-55594-5)
Supplement: Supplementary file 1 — Supplementary Figure 1. [file 41598_2024_55594_MOESM1_ESM.docx]

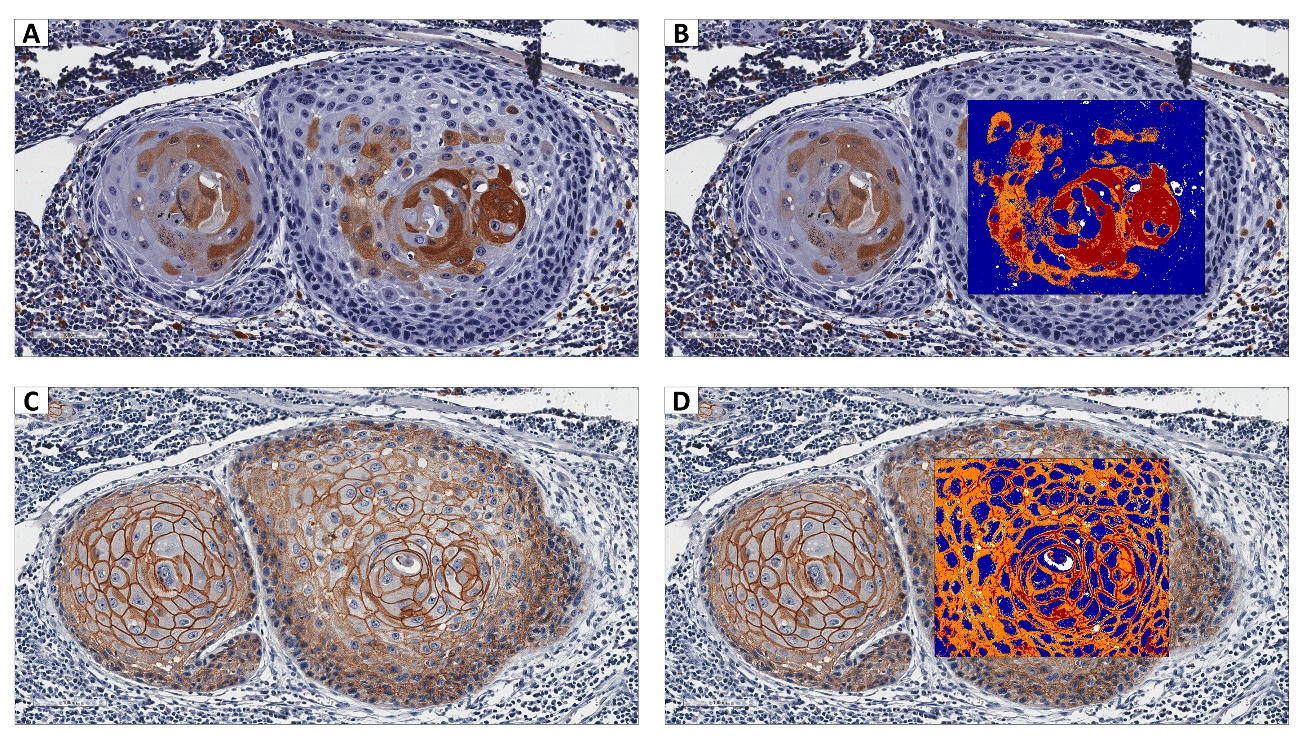


**Supplementary Figure 1.** **Immunostaining automatic analysis.** Shows the standard model of analyses by Pixel Count V9 (Aperio Technologies Inc, Vista, CA, USA), since the blue color represents negative cells, the yellow color represents the low intensity of positive cells, orange color represents the regular intensity of positive cells and the red color represents the robust intensity of positive cells.
