# Supplementary material for: CSChighE-cadherinlow immunohistochemistry panel predicts poor prognosis in oral squamous cell carcinoma
Source: Sci Rep. 2024 May 8;14:10583. doi: 10.1038/s41598-024-55594-5 (PMC11078993; doi:10.1038/s41598-024-55594-5)
Supplement: Supplementary file 2 — Supplementary Figure 2. [file 41598_2024_55594_MOESM2_ESM.docx]

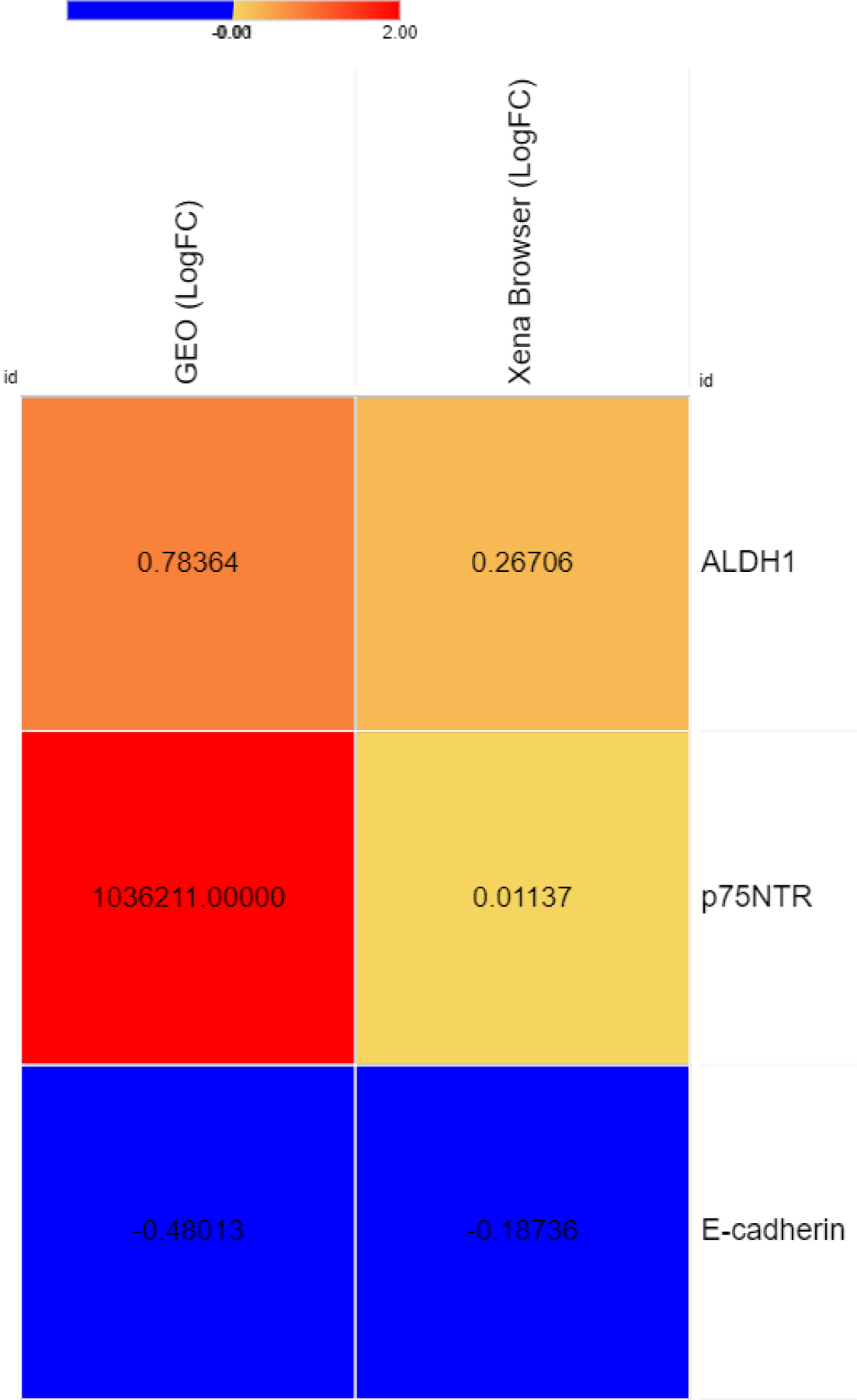


**Supplementary Figure 2.** **Heatmap showing gene expression direction of markers in two public datasets.** The regulation is shown in blue for decreased expression and shades ranging from yellow to red for increased expression.
